# Measuring state and trait anxiety in Turkish adolescents: development and validation of the six-item short forms of the state–trait anxiety inventory

**DOI:** 10.3389/fpsyg.2026.1955613

**Published:** 2026-10-09

**Authors:** Asiye Arıcı Gürbüz, Berhan Akdağ

**Affiliations:** 1 Department of Child and Adolescent Psychiatry, Dr. Ekrem Tok Mental Health and Diseases Hospital, Adana, Türkiye; 2 Department of Child and Adolescent Psychiatry, Silifke State Hospital, Mersin, Türkiye

**Keywords:** adolescent, anxiety, reliability, short form, state–trait anxiety inventory, validity

## Abstract

**Objective:**

Although the State–Trait Anxiety Inventory (STAI) is a well-established instrument for anxiety assessment, its length may limit feasibility in large-scale, longitudinal, and time-sensitive research settings. Existing abbreviated forms were mainly created for adults worldwide, including Türkiye. Accordingly, this study aimed to develop and psychometrically evaluate abbreviated versions of the STAI-State (STAI-S) and STAI-Trait (STAI-T) for Turkish adolescents.

**Method:**

A two-phase psychometric validation design was implemented across two independent cohorts. Exploratory factor analysis (EFA) was initially conducted in Sample 1, comprising adolescents recruited from an outpatient child and adolescent psychiatry clinic (*n* = 420), to identify the most parsimonious item configurations. Subsequently, confirmatory factor analysis (CFA) and criterion-related validity analyses were performed in Sample 2, consisting of public high school students (*n* = 277). Measurement invariance across clinical/community status, sex, and age, comparisons with alternative factor models, and receiver operating characteristic analyses against the Generalized Anxiety Disorder-7 were examined.

**Results:**

EFA supported a stable two-factor structure for both six-item scales, accounting for 53.7% of the total variance for the STAI-S and 57.3% for the STAI-T. Internal consistency was high for both abbreviated instruments (*α* = 0.804). CFA indicated mixed but broadly acceptable fit for the two-factor solutions (STAI-S: χ^2^/df = 3.66, CFI = 0.979, SRMR = 0.033, RMSEA = 0.098; STAI-T: χ^2^/df = 3.28, CFI = 0.987, SRMR = 0.028, RMSEA = 0.091). Standardized factor loadings ranged from 0.61 to 0.92 across models. Invariance of item thresholds and factor loadings held across clinical/community status, sex, and age in analyses treating the items as ordered-categorical. The shortened versions also showed a high correlation with the original 20-item scales and preserved their criterion-related performance. Concurrent validity was supported by correlations with the Generalized Anxiety Disorder-7 and the Satisfaction with Life Scale.

**Conclusion:**

The findings provide preliminary evidence supporting the reliability and structural validity of the six-item STAI-S and STAI-T in Turkish adolescents. The abbreviated forms appear suitable for research use, whereas their clinical utility requires confirmation in independent samples.

## Introduction

Anxiety disorders represent the most prevalent psychiatric conditions among children and adolescents and are associated with substantial impairments across multiple domains of functioning (Polanczyk et al., 2015). These impairments affect the capacity of young individuals to participate effectively in routine daily activities and to fulfill expected roles within family, academic, and social environments (Dickson et al., 2022). Adolescents with anxiety disorders frequently encounter considerable interpersonal difficulties, including heightened feelings of loneliness (Chen et al., 2023) and diminished social competence (Kaeppler and Erath, 2017), and heightened vulnerability to bullying victimization in cases particularly involving attention-deficit/hyperactivity disorder (Pityaratstian and Prasartpornsirichoke, 2023). Academic functioning is likewise adversely affected, with anxiety associated with increased school absenteeism (Finning et al., 2019) and with impairments in academic functioning (de Lijster et al., 2018; Kajastus et al., 2024). Moreover, anxiety disorders during childhood and adolescence are linked to significantly poorer quality of life across diverse psychosocial domains relative to healthy peers (Dickson et al., 2024). The broader societal burden of these disorders is also considerable, with healthcare expenditures and indirect costs, including caregiver productivity loss, exceeding those observed among children without psychiatric diagnoses (Pollard et al., 2023).

The Spielberger State–Trait Anxiety Inventory (STAI) remains one of the most extensively utilized instruments for the assessment of anxiety in both clinical and non-clinical populations (Dümmler et al., 2025). The instrument comprises two distinct 20-item subscales: the State Anxiety subscale (STAI-S), which assesses transient emotional states characterized by tension, apprehension, and heightened autonomic arousal, and the Trait Anxiety subscale (STAI-T), which evaluates a relatively stable predisposition to perceive situations as threatening (Fioravanti-Bastos et al., 2011). Each item is rated on a 4-point Likert scale, with higher scores indicating greater anxiety severity. The STAI-S includes 10 reverse-scored items, whereas the STAI-T contains seven reverse-scored items. Since its introduction, the STAI has demonstrated robust applicability across diverse cultural and clinical settings, including Turkish populations (Öner and Le Compte, 1998). Nevertheless, the length of the instrument has been criticized as burdensome for both respondents and clinicians, thereby limiting its utility in large-scale, longitudinal, and time-constrained assessment settings. Prolonged questionnaires may also increase respondent fatigue, potentially diminishing response accuracy and overall data quality.

To address these limitations, several brief versions of the STAI have been developed. For instance, Marteau and Bekker (1992) developed a six-item abbreviated version of the STAI-S, but not the STAI-T, selecting items based on corrected item–total correlations within a sample of adult women awaiting surgical procedures. Fioravanti-Bastos et al. (2011) developed the six-item short forms of the STAI-S and STAI-T in a large sample of Brazilian adolescents and adults, maintaining a balance between positive and reverse items. Du et al. (2022) generated the six-item Chinese version of the STAI-S and STAI-T for adult respondents aged 18–40, while Valente et al. (2025) recently validated a shortened STAI-Y in Italian young adults by examining measurement invariance. Additionally, Zsido et al. (2020) established five-item versions of the STAI-S and STAI-T among Hungarian adults. They excluded reverse-scored items and applied item response theory to the remaining items, which indicate the presence of anxiety. Subsequently, Döner et al. (2024) adapted these five-item forms into Turkish using a non-clinical adult sample of students and staff from a university’s central campus. The factor loadings for these short forms of the STAI-S and STAI-T ranged from 0.706 to 0.835 and 0.694 to 0.810, respectively. The internal consistency was also satisfactory, with Cronbach’s alpha values of 0.838 and 0.837 (Döner et al., 2024). However, these five-item forms of STAI-S and STAI-T have not been validated for Turkish adolescents or clinical populations. Furthermore, they exclusively included positive items, which indicate the presence of anxiety, but did not incorporate reverse-scored items. Therefore, the present study aimed to develop concise versions of the STAI-S and STAI-T for use in Turkish adolescents while maintaining a balance between positive and reverse items. It also aimed to test measurement invariance across clinical and community samples, compare alternative factor models, and estimate diagnostic accuracy of the abbreviated forms.

## Methods

### Participants and procedure

The study was conducted using two independent participant samples. The first sample (Sample 1, *n* = 420) was used for item selection and exploratory factor analysis. This cohort comprised adolescents aged 11–17 years who attended an outpatient child and adolescent psychiatry clinic. The mean age was 14.40 years (SD = 2.04), and 56.4% (*n* = 237) of participants were female. Overall, 97.9% (*n* = 411) were receiving psychiatric follow-up for at least one diagnosed condition. The most common diagnosis was attention-deficit/hyperactivity disorder (57.9%, *n* = 243), followed by generalized anxiety disorder (30.0%, *n* = 126), oppositional defiant disorder (25.5%, *n* = 107), depressive disorder (21.7%, *n* = 91), and exam anxiety (12.4%, *n* = 52). Comorbid psychiatric diagnoses were present in 54.5% of participants (*n* = 229). Exclusion criteria included a clinical diagnosis of psychosis, autism spectrum disorder, intellectual disability, substance use, or refusal to participate in the study.

The second sample (Sample 2, *n* = 277) consisted of adolescents aged 13–18 years enrolled in two public high schools in Adana. This cohort was used to evaluate the factor structure of the abbreviated STAI through confirmatory factor analysis. The mean age was 15.60 years (SD = 1.08), and 52.7% (*n* = 146) of participants were female. Among the participants, 13.4% (*n* = 37) were receiving psychiatric follow-up for at least one diagnosed condition, and 7.6% (*n* = 21) reported a chronic medical condition.

Ethical approval was obtained from the Adana Training and Research Hospital Scientific Research Ethics Committee (Approval no. 11.09.2024/139). All study procedures were conducted in accordance with the Declaration of Helsinki. Written informed consent was obtained from the parents or legal guardians of all participants, and written informed assent was obtained from every adolescent before participation. Adolescents whose parents had consented, but who did not themselves assent, were not enrolled. Participants completed questionnaires anonymously in a single session under the supervision of a clinician (Sample 1) or a researcher and class teacher (Sample 2).

### Measures

In addition to the STAI-S and STAI-T, all adolescents completed the Generalized Anxiety Disorder-7 (GAD-7) (Konkan et al., 2013; Spitzer et al., 2006) and the Satisfaction with Life Scale (SWLS) (Dağlı and Baysal, 2016; Diener et al., 1985) to assess the external validity of the abbreviated forms across both samples.

**The State–Trait Anxiety Inventory (STAI)** comprises two distinct 20-item subscales: the STAI-S and the STAI-T, as mentioned above (Spielberger et al., 1983). Each item is rated on a 4-point Likert scale ranging from “1 = not at all / almost never to “4 = very much so / almost always.” The total score can range from 20 to 80, with higher scores indicating greater anxiety severity. Öner and Le Compte (1998) adapted the STAI-S and STAI-T for the Turkish population. Although the Turkish adaptation was not originally conducted on an adolescent-only normative sample, the STAI-S and STAI-T have been utilized extensively in Turkish adolescents. In this study, no additional linguistic adaptation of item wording was conducted; the published Turkish items were administered verbatim, and the administering clinician or researcher observed comprehension and was available throughout to clarify item meaning.

**The Generalized Anxiety Disorder-7 (GAD-7)** is a seven-item self-report instrument developed to evaluate the severity of anxiety over the past 2 weeks (Konkan et al., 2013). Each item is scored on a 4-point Likert scale, from “0 = not at all” to “3 = nearly every day.” The total score ranges from 0 to 21, with higher scores representing more severe anxiety symptoms. The GAD-7 has been confirmed as a reliable and valid tool for use within the Turkish population (Spitzer et al., 2006). In the original article, a cut-off value of 10 for the total score was found to be the threshold for diagnosing GAD (Spitzer et al., 2006), whereas this score was found to be 8 in the Turkish sample (Konkan et al., 2013). In this study, the internal consistency was strong across the total sample (Cronbach’s α = 0.892, McDonald’s ω = 0.894).

**The Satisfaction with Life Scale (SWLS)** is a five-item measure assessing overall life satisfaction among respondents (Dağlı and Baysal, 2016). Respondents rate the items on a 5-point Likert scale, from “1 = strongly disagree” to “5 = strongly agree.” Total scores range from 5 to 25, with higher scores reflecting greater life satisfaction. The SWLS has demonstrated strong reliability and validity in Turkish classroom teachers (Diener et al., 1985). This study showed high internal consistency, with an α of 0.865 and a ω of 0.869.

### Scoring of the abbreviated forms

Both abbreviated instruments, the STAI-S-6 and STAI-T-6, consist of six items each, rated on the original 4-point Likert scale. Each version includes three items that directly measure anxiety and three that are reverse-scored to reflect the absence of anxiety. The STAI-S-6 includes Items 3, 10, 14, 16, 17, and 20 from the original STAI-S, with Items 10, 16, and 20 being reverse-scored. Similarly, the STAI-T-6 comprises Items 1, 10, 15, 16, 17, and 20 from the original STAI-T, with Items 1, 10, and 16 reverse-scored. Reverse-scored items are recoded by subtracting the raw response from 5 before summing. The total score, ranging from 6 to 24, is simply the sum of the six recoded items, with higher scores indicating greater state or trait anxiety.

### Statistical analysis

All statistical analyses were performed using Jamovi version 2.6.44 and JASP version 0.19.3. All questionnaires in both samples were fully completed, with no missing responses. Therefore, analyses were performed on complete cases only, and no data imputation was necessary. Item distributions were inspected before analysis; absolute skewness and kurtosis values for all retained items were below 1.0 and 1.5, respectively, indicating no severe departures from normality, and no item showed floor or ceiling concentrations exceeding the conventional 80% threshold. Item selection was undertaken according to the methodology proposed by Marteau and Bekker (1992). Specifically, STAI-S and STAI-T items were ranked according to their corrected item–total correlations. Based on these rankings, equal numbers of items representing the “absence of anxiety” (reverse-scored items) and the “presence of anxiety” were selected to construct abbreviated 10-, 8-, and 6-item versions of each scale (Marteau and Bekker, 1992; Du et al., 2022; Valente et al., 2025). This procedure is consistent with Spielberger’s recommendation (Spielberger et al., 1983) that balanced representation of anxiety-present and anxiety-absent items enhances scale reliability and measurement stability.

Marteau and Bekker (1992) applied this procedure only to the STAI-S; however, the present study extends the same methodological approach to both the STAI-S and the STAI-T. Item selection was not based on the item–total correlation ranking alone. Three additional criteria were applied. First, the balanced representation requirement described above ensured that both the presence and the absence poles of the construct, which constitute the two content domains of Spielberger’s model, were equally represented. Second, candidate items were required to show a primary loading of at least 0.40 on the intended factor in the exploratory solution and no cross-loading above 0.30, so that each retained item unambiguously represented one content domain. Third, the retained sets were inspected for content coverage of the somatic-tension, apprehension, and calm/self-confidence facets of the parent scales, so that no facet was represented exclusively.

No formal *a priori* power analysis was conducted, since the study was designed around the available consecutive clinical and school cohorts. *Post hoc*, the achieved sample sizes are adequate by conventional standards. For the exploratory phase, the ratio of participants to items was 21:1 for the 20-item scales and 70:1 for the 6-item scales, well above the commonly recommended minima of 5:1 to 10:1, and the absolute sample size of 420 exceeds the threshold at which factor recovery is generally stable given communalities in the moderate-to-high range observed here (MacCallum et al., 1996). For the confirmatory phase, *N* = 277 corresponds to approximately 40 observations per free parameter in the six-item two-factor models, comfortably above the 10:1 ratio typically recommended. One limitation should nonetheless be acknowledged explicitly: because the six-item models have only 8 degrees of freedom (df), statistical power for the test of close fit is low (1 − *β* = 0.34 at *N* = 277; approximately 950 participants would be required for power of 0.80). This is a general property of low-df models rather than a feature of the present data, and it is the principal reason the root mean square error of approximation (RMSEA) is interpreted cautiously below.

Subsequently, exploratory factor analysis (EFA) and confirmatory factor analysis (CFA) were conducted to evaluate the factor structure of the abbreviated forms. EFA was performed using principal axis factoring with promax rotation. Sampling adequacy was verified with the Kaiser–Meyer–Olkin (KMO) measure and Bartlett’s test of sphericity, and the number of factors was determined jointly by eigenvalues greater than one and inspection of the scree plot. CFA was conducted using the robust weighted least squares mean and variance adjusted (WLSMV) estimator, which is appropriate for ordered-categorical indicators and operates on the polychoric correlation matrix. Model fit was assessed using the χ^2^ statistic and RMSEA. A non-significant χ^2^ statistic and an RMSEA value below 0.08 were considered indicative of acceptable model fit (MacCallum et al., 1996; Browne and Cudeck, 1992). Exact χ^2^ values with their degrees of freedom and *p* values, the RMSEA point estimate with its 90% confidence interval, and the test of close fit (p_close_) are reported throughout. Given the sensitivity of the χ^2^ statistic to sample size, the χ^2^/*df* ratio was also examined. Values ≤ 2 were interpreted as reflecting excellent fit, whereas values between 2 and 5 were considered indicative of acceptable fit (Hu and Bentler, 1999). Additional fit indices included the comparative fit index (CFI), Tucker–Lewis index (TLI), and goodness-of-fit index (GFI), together with the standardized root mean square residual (SRMR), for which values below 0.08 indicate acceptable fit. Model fit was considered satisfactory when CFI, TLI, and GFI values were ≥ 0.95 (Hu and Bentler, 1999). For each scale, the hypothesized two-factor solution was compared with a competing unidimensional model estimated on the same data with the same estimator, and unstandardized and standardized parameter estimates with robust standard errors, z values, *p* values, and 95% confidence intervals, together with standardized residual correlations and the factor covariance, are reported in full.

Because item selection was carried out in a predominantly clinical sample whereas confirmation was carried out in a community sample, measurement invariance was formally tested across the two cohorts in the combined dataset (*N* = 697), and additionally across sex and across age (median split at 14 years). Consistent with the treatment of the four-category STAI items as ordered-categorical indicators in the primary CFA, the invariance analyses were conducted within the same ordinal framework: the WLSMV estimator was applied to the group-specific thresholds and polychoric correlation matrices under the delta parameterization, and the items were therefore not treated as continuous. For ordered-categorical indicators, item measurement parameters are thresholds rather than continuous-variable intercepts, and thresholds and loadings are not separately identified when scale factors are freely estimated; invariance was therefore tested in the sequence recommended for ordinal data (Millsap and Yun-Tein, 2004; Wu and Estabrook, 2016; Svetina et al., 2020). Three nested models were compared: a configural model, in which thresholds, loadings and the factor correlation were free in both groups; a threshold-and-loading invariance model, in which thresholds and loadings were constrained to equality across groups while scale factors, factor variances and latent means were freely estimated in the second group; and a residual invariance model, which additionally constrained the scale factors, and hence the residual variances, to equality. Identification followed Millsap and Yun-Tein (2004), with the first group as reference (latent means fixed at zero, factor variances and scale factors fixed at unity). Invariance was retained when the change in CFI was ≤ 0.010 and the change in RMSEA was ≤ 0.015 (Cheung and Rensvold, 2002; Chen, 2007); the χ^2^ difference test is reported alongside but was not treated as decisive given its sensitivity to sample size. Where an invariance step failed, partial invariance was explored by sequentially releasing individual item parameters. For comparability with the earlier literature, the corresponding continuous-variable sequence (configural, equal loadings, equal loadings and intercepts) was also estimated by normal-theory maximum likelihood with the item scores treated as continuous; those results are reported as a sensitivity analysis in Supplementary Table S1 and are not the basis of any conclusion drawn here.

Pearson correlation coefficients were calculated to examine associations between the abbreviated forms and the corresponding 20-item STAI-S and STAI-T scales. Correlations exceeding 0.90 were considered indicative of strong proportionality between the short and full-length versions (Kline, 2000). Because the retained items are themselves contained within the parent scales, these coefficients are inflated by item overlap and are not independent evidence of validity; each abbreviated form was therefore additionally correlated with the sum of the 14 (or 12, or 10) non-overlapping items of its parent scale, which provides a part–whole corrected index of how well the short form represents the content it does not itself contain. Internal consistency reliability was evaluated using Cronbach’s alpha (α) and McDonald’s omega (ω). Values of ≥ 0.70 for both α and ω were considered indicative of acceptable reliability based on established recommendations in the literature (Tavakol and Dennick, 2011). Composite reliability (CR) and average variance extracted (AVE) were computed from the standardized CFA loadings, with AVE ≥ 0.50 and CR ≥ 0.70 taken as evidence of convergent validity, and discriminant validity evaluated with the Fornell–Larcker criterion (Fornell and Larcker, 1981). Finally, receiver operating characteristic (ROC) analyses were conducted to compare the accuracy of the abbreviated forms and the 20-item original scales in identifying both adolescents with a GAD-7 score of 8 or above and 10 or above. Areas under the curve (AUC) are reported with 95% confidence intervals computed by the DeLong method (DeLong et al., 1988), and optimal thresholds were identified by maximizing the Youden index, with sensitivity, specificity, positive and negative predictive values reported at that threshold. Statistical significance was set at *p* < 0.05 (two-tailed) throughout.

## Results

### Development and exploratory analyses of the short forms (Sample 1)

To develop abbreviated versions of the STAI-S and STAI-T, initial EFAs were conducted using the original 20-item scales in the outpatient sample (n = 420). Principal axis factoring with promax rotation was applied to both instruments. Preliminary analyses indicated that the data were well suited for factor analysis. The Kaiser–Meyer–Olkin (KMO) measure demonstrated excellent sampling adequacy (0.914 for STAI-S and 0.902 for STAI-T), whereas Bartlett’s Test of Sphericity confirmed the factorability of the correlation matrices [χ^2^ (190) = 3393.94 for STAI-S; χ^2^ (190) = 2794.62 for STAI-T; both *p* < 0.001]. For both scales, eigenvalues and inspection of the scree plots consistently supported a two-factor solution.

The 20-item STAI-S explained 40.9% of the total variance (Factor 1: 23.9%, 10 items representing “absence of anxiety”; Factor 2: 17.0%, 9 items representing “presence of anxiety”). All factor loadings exceeded 0.40 (range, 0.401–0.823), with the exception of Item 6 (0.388). The 20-item STAI-T accounted for 37.2% of the total variance (Factor 1: 21.8%, 12 items representing “presence of anxiety”; Factor 2: 15.4%, 6 items representing “absence of anxiety”). Factor loadings ranged from 0.410 to 0.816, with minor exceptions for Item 2 (0.391) and Item 7 (0.370). No substantial cross-loadings were identified for either scale.

Following these preliminary analyses, items were selected using the psychometric procedure proposed by Marteau and Bekker (1992). Items were ranked according to their corrected item–total correlations (Table 1), and equal numbers of reverse-scored (“absence of anxiety”) and directly scored (“presence of anxiety”) items were retained to construct abbreviated 10-, 8-, and 6-item versions.

**Table 1:** The corrected item–total correlations for the items of STAI-S and STAI-T (Sample 1, *n* = 420).

Items	STAI-S	STAI-T
Item 1	0.558*	0.524*
Item 2	0.468*	0.394
Item 3	0.516	0.434
Item 4	0.368	0.375
Item 5	0.570*	0.369
Item 6	0.419	0.456*
Item 7	0.444	0.294*
Item 8	0.468*	0.517
Item 9	0.508	0.593
Item 10	0.688*	0.561*
Item 11	0.492*	0.522
Item 12	0.515	0.443
Item 13	0.470	0.349*
Item 14	0.592	0.341
Item 15	0.670*	0.630
Item 16	0.686*	0.479*
Item 17	0.557	0.644
Item 18	0.068	0.607
Item 19	0.535*	0.386*
Item 20	0.689*	0.625

*Reverse-coded items.

Subsequent EFAs and reliability analyses were conducted to identify the most parsimonious configuration while preserving the original two-factor structure. Based on these findings, the six-item versions emerged as the optimal abbreviated forms for both inventories according to the following psychometric indicators:

The six-item STAI-S demonstrated adequate factorability (KMO = 0.797; Bartlett’s χ^2^ (15) = 788.99, *p* < 0.001) and explained 53.7% of the total variance (Factor 1 = 30.8%, Factor 2 = 22.9%). Standardized factor loadings ranged from 0.402 to 0.893 (Table 2). Internal consistency was strong (α = 0.804, ω = 0.805), and the abbreviated scale exhibited a very strong correlation with the original 20-item version (*r* = 0.949, *p* < 0.001).

**Table 2:** Results from the exploratory factor analysis of the short versions of the STAI-S (Sample 1, *n* = 420).

	STAI-S (10 items)		STAI-S (8 items)		STAI-S (6 items)	
	Factor 1	Factor 2	Factor 1	Factor 2	Factor 1	Factor 2
Item 20*	0.777		0.790		0.801	
Item 10*	0.780		0.799		0.770	
Item 16*	0.703		0.724		0.761	
Item 15*	0.805		0.808		—	—
Item 5*	0.731		—	—	—	—
Item 14		0.869		0.852		0.893
Item 17		0.433		.364ᵃ		0.402
Item 3		0.690		0.688		0.659
Item 12		0.761		0.744	—	—
Item 9		0.365ᵃ	—	—	—	—
Eigenvalue	4.56	1.44	3.91	1.31	3.03	1.06
Kaiser–Meyer–Olkin measure	0.888		0.865		0.797	
Sig. of Bartlett’s test	<0.001		<0.001		<0.001	
Total variance explained	50.7%		54.4%		53.7%	

*Reverse-coded items. ᵃItems with factor loadings below the threshold of 0.40. Factor 1 represents the “absence of anxiety” domain, consisting of reverse-coded items, while Factor 2 pertains to the “presence of anxiety” domain, which includes directly scored items.

The six-item STAI-T demonstrated satisfactory structural characteristics (KMO = 0.777; Bartlett’s χ^2^ (15) = 888.03, *P* < 0.001), accounting for 57.3% of the total variance (Factor 1 = 31.9%, Factor 2 = 25.3%). Factor loadings ranged from 0.469 to 0.935, with no cross-loadings exceeding 0.30 (Table 3). Reliability indices were similarly strong (*α* = 0.804, *ω* = 0.809), and the abbreviated form demonstrated a high correlation with the original scale (*r* = 0.895, *P* < 0.001).

**Table 3:** Results from the exploratory factor analysis of the short versions of the STAI-T (Sample 1, *n* = 420).

	STAI-T (10 items)		STAI-T (8 items)		STAI-T (6 items)	
	Factor 1	Factor 2	Factor 1	Factor 2	Factor 1	Factor 2
Item 10*	0.863		0.888		0.935	
Item 1*	0.771		0.781		0.702	
Item 16*	0.747		0.704		0.707	
Item 6*	0.581		0.589		—	—
Item 19*	0.468		—	—	—	—
Item 17		0.775		0.774		0.826
Item 15		0.492		0.500		0.469
Item 20		0.781		0.772		0.791
Item 18		0.744		0.790	—	—
Item 9		0.704	—	—	—	—
Eigenvalue	4.28	1.68	3.78	1.44	3.07	1.17
Kaiser–Meyer–Olkin measure	0.872		0.843		0.777	
Sig. of Bartlett’s test	<0.001		<0.001		<0.001	
Total variance explained	50.2%		54.4%		57.3%	

*Reverse-coded items. Factor 1 represents the “absence of anxiety” domain, consisting of reverse-coded items, while Factor 2 pertains to the “presence of anxiety” domain, which includes directly scored items.

Given that the 8- and 10-item forms demonstrated nominally higher internal consistency, the selection of the six-item configuration was explicitly evaluated against four criteria (Table 4). First, for reliability, the relevant comparison is not the raw α but the α expected for a scale of that length. Utilizing the mean inter-item correlation of the 20-item scales, the Spearman–Brown prediction for a randomly chosen six-item subset is *α* = 0.715 for the STAI-S and 0.679 for the STAI-T in Sample 1. The observed values of 0.804 for both forms significantly surpass these expectations, indicating that the selected items are markedly more efficient per item than the average item in the 20-item scale. Secondly, regarding construct representation, the part–whole corrected correlations with the non-overlapping remainder of the 20-item scale were actually highest for the six-item forms (Sample 2: STAI-S-6 = 0.833 compared to 0.798 and 0.808 for the 8- and 10-item forms; STAI-T-6 = 0.749 versus 0.727 and 0.741). The additional items retained in the longer forms therefore contributed minimal unique information about the content they omit. Thirdly, in terms of criterion-related validity, the six-item forms performed comparably to the longer forms and to the 20-item scales (Table 4); the STAI-T-6 correlated with the GAD-7 at *r* = 0.645 in Sample 2, which is essentially indistinguishable from the 20-item STAI-T (*r* = 0.661). Fourth, in terms of practical utility, the six-item forms reduce administration burden by 70% while maintaining the balanced 3:3 representation of the presence and absence content poles. This balance is only achieved in the 8- and 10-item configurations by adding items with weaker or less distinctive loadings. In summary, the six-item forms provide the most advantageous balance among brevity, reliability, content representation, and criterion validity, rather than being selected solely for brevity.

**Table 4:** Psychometric comparison of the 6-, 8-, and 10-item abbreviated forms with the 20-item scales.

Form	*k*	*α*	*ω*	*r* with the 20-item scale	*r* with remainder	*r* with GAD-7	*r* with SWLS
Sample 1 (*n* = 420)							
STAI-S-6	6	0.804	0.805	0.949	0.879	0.574	−0.533
STAI-S-8	8	0.850	0.851	0.956	0.853	0.584	−0.519
STAI-S-10	10	0.866	0.867	0.971	0.849	0.608	−0.540
STAI-S-20	20	0.896	0.896	—	—	0.605	−0.557
STAI-T-6	6	0.804	0.809	0.895	0.752	0.645	−0.581
STAI-T-8	8	0.837	0.840	0.916	0.719	0.678	−0.586
STAI-T-10	10	0.847	0.850	0.944	0.726	0.683	−0.594
STAI-T-20	20	0.876	0.878	—	—	0.681	−0.536
Sample 2 (*n* = 277)							
STAI-S-6	6	0.764	0.765	0.925	0.833	0.461	−0.508
STAI-S-8	8	0.822	0.822	0.935	0.798	0.457	−0.490
STAI-S-10	10	0.850	0.850	0.960	0.808	0.496	−0.518
STAI-S-20	20	0.888	0.889	—	—	0.530	−0.559
STAI-T-6	6	0.779	0.784	0.884	0.749	0.645	−0.550
STAI-T-8	8	0.809	0.813	0.906	0.727	0.667	−0.554
STAI-T-10	10	0.832	0.834	0.938	0.741	0.686	−0.546
STAI-T-20	20	0.886	0.887	—	—	0.661	−0.510

k = number of items. *α* = Cronbach’s alpha, *ω* = McDonald’s omega, *r* with the 20-item scale = correlation with the corresponding 20-item scale; this coefficient is inflated by part–whole overlap. *r* with remainder = correlation with the sum of the parent-scale items that are not contained in the abbreviated form, and is therefore free of item overlap. All correlations were significant at *p* < 0.001. For reference, the Spearman–Brown expectation for a randomly selected six-item subset, computed from the mean inter-item correlation of the 20-item parent scale, is *α* = 0.715 (STAI-S) and 0.679 (STAI-T) in Sample 1 and 0.703 and 0.699 in Sample 2; the observed values for the retained six-item forms exceed these expectations in every case.

### Structural confirmation and validity testing (sample 2)

To evaluate the structural stability of the six-item STAI-S and STAI-T, CFAs were conducted in an independent school-based sample (*n* = 277). As shown in Figures 1, 2 and Table 5, both abbreviated forms retained the two-factor structure, although model fit was mixed rather than uniformly satisfactory:

**Figure 1:**
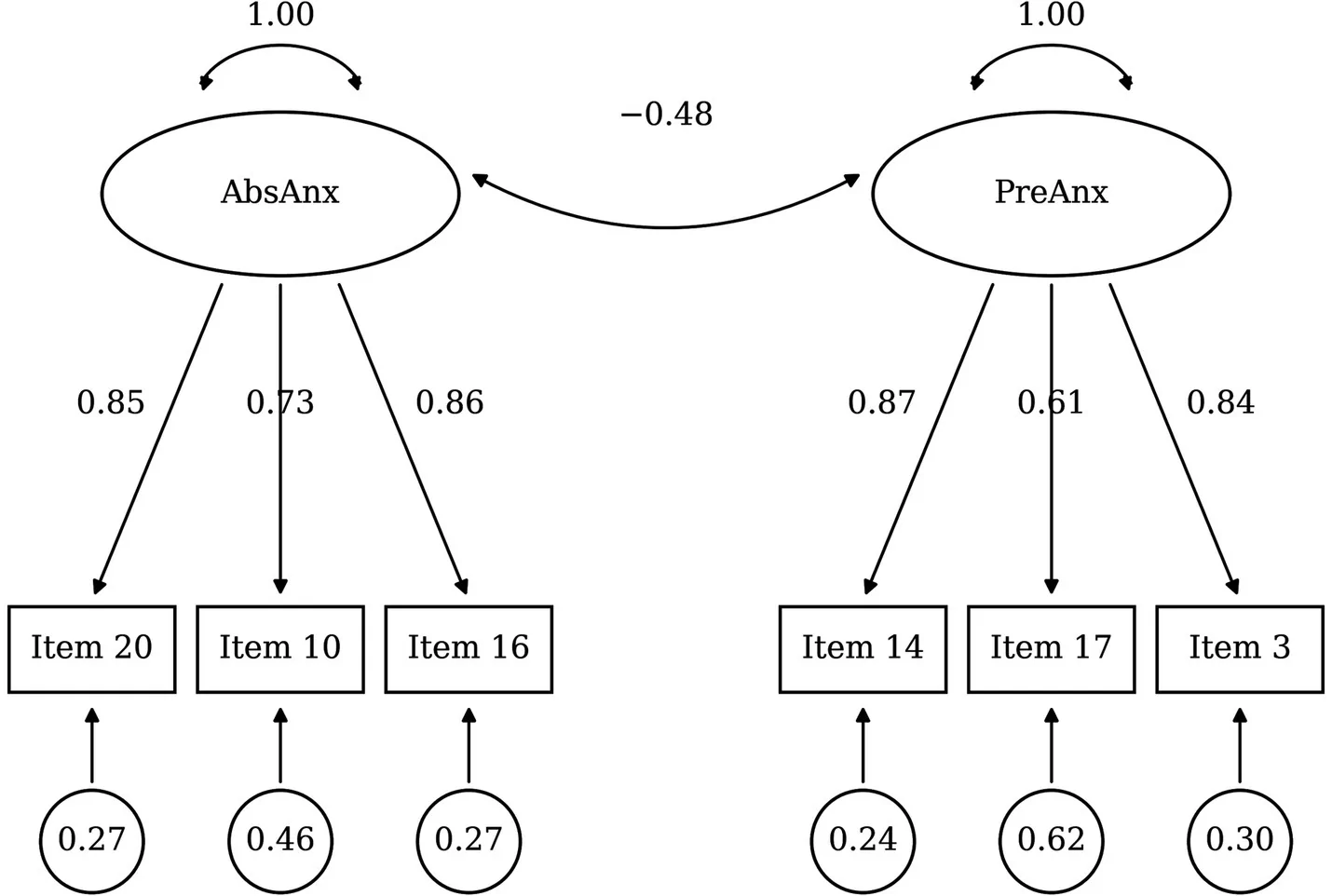
Standardized factor loadings of confirmatory factor analyses for the six-item STAI-S (Sample 2, *n* = 277). All coefficients are standardized. AbsAnx = absence of anxiety, the latent factor defined by the reverse-coded items (Items 10, 16, and 20), which describe calm, comfort and wellbeing; PreAnx = presence of anxiety, the latent factor defined by the directly scored items (Items 3, 14, and 17), which describe tension, apprehension and nervousness. Rectangles denote observed items, ellipses denote latent factors, and circles denote standardized residual variances. The value on the curved double-headed arrow is the correlation between the two factors, estimated on the raw item responses; it is negative because the AbsAnx items are scored in the opposite direction to the total score, and corresponds to a positive association of 0.48 once these items are reverse-recoded. The value 1.00 above each factor indicates that its variance was fixed at unity for identification. Corresponding standard errors, *p*-values, and confidence intervals are reported in Table 5.

**Figure 2:**
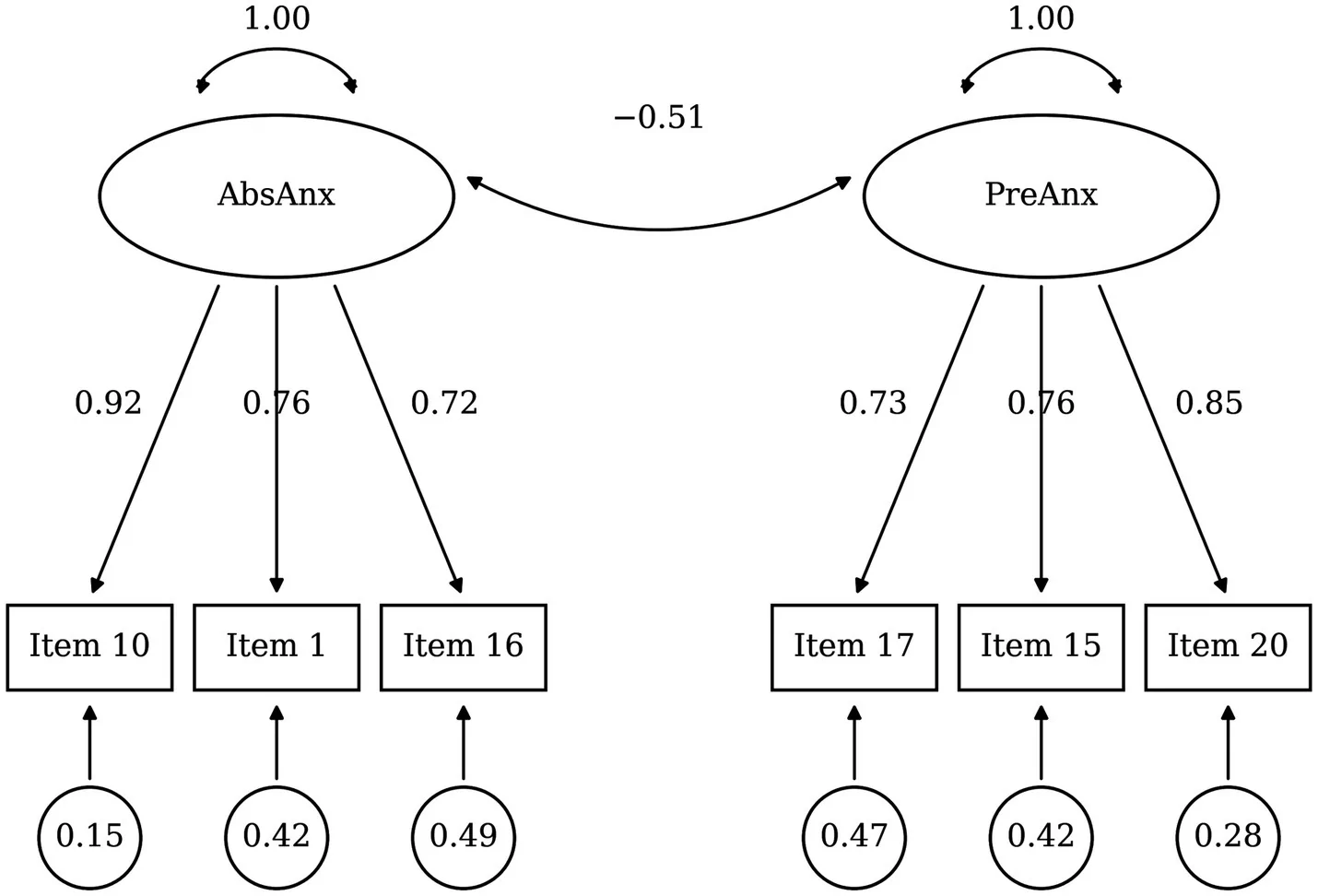
Standardized factor loadings of confirmatory factor analyses for the six-item STAI-T (Sample 2, *n* = 277). All coefficients are standardized. AbsAnx = absence of anxiety, the latent factor defined by the reverse-coded items (Items 1, 10, and 16), which describe calm, self-confidence and contentment; PreAnx = presence of anxiety, the latent factor defined by the directly scored items (Items 15, 17, and 20), which describe worry, tension and rumination. Rectangles denote observed items, ellipses denote latent factors, and circles denote standardized residual variances. The value on the curved double-headed arrow is the correlation between the two factors, estimated on the raw item responses; it is negative because the AbsAnx items are scored in the opposite direction to the total score, and corresponds to a positive association of 0.51 once these items are reverse-recoded. The value 1.00 above each factor indicates that its variance was fixed at unity for identification. Corresponding standard errors, *p*-values, and confidence intervals are reported in Table 5.

**Table 5:** Confirmatory factor analysis parameter estimates for the six-item STAI-S and STAI-T (Sample 2, *n* = 277).

Factor / Item	λ	SE	z	p	95% CI	R^2^	δ	Item mean (SD)
STAI-S-6								
AbsAnx (absence of anxiety)								
Item 20	0.854	0.037	23.24	<0.001	[0.782, 0.926]	0.729	0.271	2.11 (0.99)
Item 10	0.732	0.057	12.74	<0.001	[0.619, 0.845]	0.536	0.464	2.27 (1.00)
Item 16	0.856	0.047	18.30	<0.001	[0.764, 0.947]	0.732	0.268	2.18 (0.99)
PreAnx (presence of anxiety)								
Item 14	0.870	0.045	19.37	<0.001	[0.782, 0.958]	0.757	0.243	1.99 (0.94)
Item 17	0.614	0.063	9.79	<0.001	[0.491, 0.737]	0.377	0.623	2.32 (0.99)
Item 3	0.836	0.056	15.03	<0.001	[0.727, 0.945]	0.698	0.302	2.09 (0.95)
AbsAnx ↔ PreAnx	0.482	0.086	5.61	<0.001	[0.313, 0.650]	—	—	—
STAI-T-6								
AbsAnx (absence of anxiety)								
Item 10	0.924	0.043	21.68	<0.001	[0.840, 1.007]	0.853	0.147	2.34 (1.02)
Item 1	0.760	0.047	16.10	<0.001	[0.668, 0.853]	0.578	0.422	2.20 (0.94)
Item 16	0.716	0.060	11.89	<0.001	[0.598, 0.835]	0.513	0.487	2.31 (0.98)
PreAnx (presence of anxiety)								
Item 17	0.727	0.055	13.30	<0.001	[0.620, 0.835]	0.529	0.471	2.34 (1.01)
Item 15	0.764	0.048	15.82	<0.001	[0.669, 0.859]	0.584	0.416	2.28 (0.99)
Item 20	0.846	0.042	20.06	<0.001	[0.764, 0.929]	0.716	0.284	2.30 (0.99)
AbsAnx ↔ PreAnx	0.506	0.080	6.33	<0.001	[0.349, 0.663]	—	—	—

Estimates were obtained with the robust weighted least squares mean- and variance-adjusted (WLSMV) estimator applied to the polychoric correlation matrix. Because latent variances are fixed at unity and indicators are standardized under this parameterization, the unstandardized and standardized loadings are identical; λ therefore denotes the standardized factor loading. SE = robust standard error; CI = confidence interval; *R*^2^ = proportion of item variance explained by the factor; δ = standardized residual variance (1 − R^2^). AbsAnx ↔ PreAnx = standardized covariance (i.e., correlation) between the two factors. Item means and standard deviations are given on the original 1–4 response metric after reverse recoding. All standardized residual correlations were below 0.10 in absolute value.

The six-item STAI-S: χ^2^ (8) = 29.28, *p* < 0.001, χ^2^/df = 3.66, RMSEA = 0.098, 90% CI [0.079, 0.109], *p*_close_ = 0.003, SRMR = 0.033, GFI = 0.996, CFI = 0.979, and TLI = 0.961. Standardized loadings ranged from 0.614 to 0.870 (Figure 1 and Table 5).

The six-item STAI-T: χ^2^ (8) = 26.24, *p* < 0.001, χ^2^/df = 3.28, RMSEA = 0.091, 90% CI [0.070, 0.103], *p*_close_ = 0.008, SRMR = 0.028, GFI = 0.997, CFI = 0.987, and TLI = 0.976. Standardized loadings ranged from 0.716 to 0.924 (Figure 2 and Table 5).

For both scales, the incremental and residual-based indices were clearly satisfactory (CFI and TLI ≥ 0.96; SRMR ≤ 0.033; GFI ≥ 0.996), whereas the RMSEA exceeded the prespecified criterion of 0.08 and its 90% confidence interval excluded values indicative of close fit. All standardized loadings were substantial and statistically significant, with 95% confidence intervals excluding zero and with the smallest lower bound at 0.491 (Table 5); the factor covariance was moderate in both models (STAI-S-6: 0.48, 95% CI [0.31, 0.65]; STAI-T-6: 0.51, 95% CI [0.35, 0.66]), confirming that the two content domains are related but distinguishable. Standardized residual correlations were small throughout (all |r| < 0.10), indicating no systematic local misfit. The evidence for the hypothesized structure is therefore mixed: strong on incremental and residual criteria, weaker on the RMSEA. As set out in the Methods, the RMSEA is estimated imprecisely in models with only 8 degrees of freedom, and power to detect close fit at this sample size was only 0.34, so this index should not be treated as decisive in either direction.

For each scale, a competing unidimensional model, in which all six items loaded on a single anxiety factor, was estimated on the same data with the same estimator. Both unidimensional models fitted markedly worse than the two-factor solutions (STAI-S-6: χ^2^ (9) = 102.10, *p* < 0.001, RMSEA = 0.193, 90% CI [0.185, 0.199], CFI = 0.919, TLI = 0.866, SRMR = 0.156; STAI-T-6: χ^2^ (9) = 93.69, *p* < 0.001, RMSEA = 0.184, 90% CI [0.175, 0.190], CFI = 0.909, TLI = 0.848, SRMR = 0.137), with every index falling well below conventional thresholds and with χ^2^ differences of 72.82 and 67.45 on a single degree of freedom (both *p* < 0.001). The two-factor solution was therefore retained for both scales (Table 6).

**Table 6:** Fit of the retained two-factor models and of competing unidimensional models (Sample 2, *n* = 277).

Model	χ^2^	df	*p*	χ^2^/df	RMSEA [90% CI]	CFI	TLI	SRMR
STAI-S-6								
Two-factor (retained)	29.28	8	<0.001	3.66	0.098 [0.079, 0.109]	0.979	0.961	0.033
One-factor	102.10	9	<0.001	11.34	0.193 [0.185, 0.199]	0.919	0.866	0.156
STAI-T-6								
Two-factor (retained)	26.24	8	<0.001	3.28	0.091 [0.070, 0.103]	0.987	0.976	0.028
One-factor	93.69	9	<0.001	10.41	0.184 [0.175, 0.190]	0.909	0.848	0.137

All models were estimated with the WLSMV estimator on the polychoric correlation matrix, with the same six indicators in each case. The two-factor models specify separate, correlated AbsAnx and PreAnx factors; the one-factor models specify a single anxiety factor. χ^2^ difference tests favored the two-factor solutions for both scales (STAI-S-6: Δχ^2^ (1) = 72.82, *p* < 0.001; STAI-T-6: Δχ^2^ (1) = 67.45, *p* < 0.001). For the retained models, the close-fit test was *p*_close_ = 0.003 (STAI-S-6) and 0.008 (STAI-T-6); with only 8 degrees of freedom, the RMSEA is estimated imprecisely, and the power for the close-fit test was 0.34.

Both abbreviated scales also demonstrated satisfactory internal consistency within the validation sample (STAI-S: α = 0.764, ω = 0.766; STAI-T: α = 0.779, ω = 0.784). CR and AVE computed from the CFA loadings likewise met conventional criteria for both factors of both scales (STAI-S-6: CR = 0.856 and 0.822, AVE = 0.666 and 0.611; STAI-T-6: CR = 0.845 and 0.824, AVE = 0.648 and 0.610). In each model, the squared factor correlation (0.23 and 0.26) was smaller than either AVE, satisfying the Fornell–Larcker criterion for discriminant validity between the presence and absence domains.

Multiple-group analyses in the combined dataset (*N* = 697), estimated within the ordinal WLSMV framework, supported configural invariance of both abbreviated forms across clinical and community status, sex, and age (Table 7). Constraining item thresholds and factor loadings to equality across groups produced negligible deterioration in fit in every comparison (ΔCFI between +0.002 and −0.004; ΔRMSEA between −0.027 and −0.004), so threshold-and-loading invariance — the ordinal counterpart of joint metric and scalar invariance — was supported throughout. The additional constraint of equal scale factors (residual invariance) was met for both scales across sex, for the STAI-T-6 across age and, marginally, for the STAI-S-6 across clinical/community status (ΔCFI = −0.009, ΔRMSEA = +0.013), but not for the STAI-T-6 across clinical/community status (ΔCFI = −0.014, ΔRMSEA = +0.018) or for the STAI-S-6 across age (ΔCFI = −0.011, ΔRMSEA = +0.019). Residual invariance is not a prerequisite for comparing latent means or associations and is rarely attained in practice; its partial failure indicates only that item-specific measurement error differs somewhat between settings and age bands.

**Table 7:** Tests of measurement invariance for the six-item STAI-S and STAI-T, estimated as ordered-categorical indicators (combined sample, *N* = 697).

Grouping / Model	χ^2^	df	CFI	TLI	RMSEA	SRMR	Δχ^2^ (Δdf)	ΔCFI	ΔRMSEA
STAI-S-6: clinical (*n* = 420) vs community (*n* = 277)									
Configural	37.28	16	0.993	0.987	0.062	0.048	—	—	—
Thresholds + loadings	64.41	30	0.989	0.989	0.057	0.052	27.12 (14), *p* = 0.019	−0.004	−0.004
+ equal residuals	98.41	36	0.980	0.983	0.071	0.054	34.00 (6), *p* < 0.001	−0.009	+0.013
STAI-T-6: clinical vs community									
Configural	51.66	16	0.988	0.978	0.080	0.053	—	—	—
Thresholds + loadings	76.09	30	0.985	0.985	0.066	0.054	24.44 (14), *p* = 0.041	−0.004	−0.014
+ equal residuals	125.33	36	0.970	0.975	0.084	0.055	49.24 (6), *p* < 0.001	−0.014	+0.018
STAI-S-6: girls (*n* = 383) vs boys (*n* = 314)									
Configural	61.77	16	0.983	0.968	0.091	0.055	—	—	—
Thresholds + loadings	71.71	30	0.984	0.984	0.063	0.060	9.94 (14), *p* = 0.767	+0.002	−0.027
+ equal residuals	83.00	36	0.982	0.985	0.061	0.057	11.29 (6), *p* = 0.080	−0.002	−0.002
STAI-T-6: girls vs boys									
Configural	50.04	16	0.988	0.977	0.078	0.055	—	—	—
Thresholds + loadings	64.90	30	0.988	0.988	0.058	0.058	14.86 (14), *p* = 0.387	−0.000	−0.020
+ equal residuals	78.08	36	0.985	0.988	0.058	0.059	13.18 (6), *p* = 0.040	−0.003	+0.000
STAI-S-6: age ≤ 14 vs > 14 years									
Configural	39.87	16	0.991	0.983	0.065	0.050	—	—	—
Thresholds + loadings	49.61	30	0.993	0.993	0.043	0.054	9.74 (14), *p* = 0.781	+0.002	−0.022
+ equal residuals	84.97	36	0.982	0.985	0.062	0.062	35.36 (6), *p* < 0.001	−0.011	+0.019
STAI-T-6: age ≤ 14 vs > 14 years									
Configural	55.90	16	0.987	0.975	0.085	0.058	—	—	—
Thresholds + loadings	64.45	30	0.989	0.989	0.057	0.059	8.54 (14), *p* = 0.859	+0.002	−0.027
+ equal residuals	70.95	36	0.988	0.990	0.053	0.059	6.50 (6), *p* = 0.369	−0.000	−0.005

Multiple-group confirmatory factor analyses estimated with the robust weighted least squares mean- and variance-adjusted (WLSMV) estimator applied to the group-specific item thresholds and polychoric correlation matrices, under the delta parameterization and the identification constraints of Millsap and Yun-Tein (White and Karr, 2025). Because the item measurement parameters of ordered-categorical indicators are thresholds rather than continuous-variable intercepts, and thresholds and loadings are not separately identified when the scale factors are free, the two sets of parameters are constrained jointly. Configural = thresholds, loadings and the factor correlation free in both groups; Thresholds + loadings = thresholds and loadings constrained equal, with scale factors, factor variances and latent means free in the second group; + equal residuals = scale factors additionally constrained. Each comparison is against the immediately preceding, less restricted model. Invariance was retained when ΔCFI ≤ 0.010 and ΔRMSEA ≤ 0.015 in absolute value. Threshold-and-loading invariance was supported in every comparison. Corresponding maximum-likelihood analyses treating the items as continuous are reported in Supplementary Table S1.

Because thresholds and loadings were invariant, latent means could be compared directly. Expressed in standard deviation units of the reference group, community adolescents did not differ appreciably from clinical adolescents on the state factors (+0.07 and +0.14) and scored slightly lower on the trait factors (−0.22 and −0.08); boys scored lower than girls on all four factors (−0.29 to −0.55); and adolescents older than 14 years scored higher than younger adolescents (+0.29 to +0.49). Equality of thresholds and loadings across all comparisons indicates that the items relate to the latent constructs in the same way and are endorsed at the same levels for a given level of anxiety, in clinical and community adolescents, in girls and boys, and in younger and older adolescents.

For comparison, the same sequence estimated by maximum likelihood with the items treated as continuous (Supplementary Table S1) reproduced the conclusion of full loading invariance but indicated non-invariance of a single STAI-T intercept across clinical and community status (ΔCFI = −0.010), which was resolved by freeing the intercept of Item 15. The ordinal analyses, which model the item thresholds directly and are the appropriate framework for four-category responses, did not reproduce this result; we therefore attribute it to the continuous approximation and regard threshold-and-loading invariance as supported for both scales across all three grouping variables.

### Criterion-related validity and diagnostic accuracy

Concurrent validity was evaluated through Pearson correlation analyses with external measures of anxiety and well-being (GAD-7 and SWLS). Consistent with theoretical expectations, the abbreviated scales demonstrated expected patterns of convergent and divergent validity:

The six-item STAI-S was positively correlated with the GAD-7 (*r* = 0.461, *p* < 0.001) and negatively correlated with the SWLS (*r* = −0.508, *p* < 0.001).

The six-item STAI-T demonstrated a similar but stronger pattern of associations, showing a positive correlation with the GAD-7 (*r* = 0.645, *p* < 0.001) and a negative correlation with the SWLS (*r* = −0.550, *p* < 0.001).

Because the six retained items are themselves part of the 20-item scales, the correlations of 0.949 and 0.895 reported above are inflated by item overlap and cannot be interpreted as independent evidence of validity. When each abbreviated form was instead correlated with the sum of the 14 non-overlapping parent-scale items, the coefficients remained substantial (Sample 1: STAI-S-6 *r* = 0.879, STAI-T-6 *r* = 0.752; Sample 2: *r* = 0.833 and 0.749, respectively), indicating that the short forms capture a large share of the variance of the content they do not themselves contain. Together with associations with the GAD-7 and the SWLS, which are fully independent of the STAI item pool, these results provide stronger evidence of external validity.

ROC curve analyses was presented in Table 8 and Figure 3. Against a GAD-7 score of 10 or above, the STAI-T-6 performed well in both cohorts (Sample 2: AUC = 0.846, 95% CI [0.796, 0.897]; Sample 1: AUC = 0.824, 95% CI [0.785, 0.863]) and was statistically indistinguishable from the 20-item STAI-T (AUC = 0.844 and 0.852, respectively). The STAI-S-6 showed more modest but acceptable discrimination (Sample 2: AUC = 0.719, 95% CI [0.655, 0.782]; Sample 1: AUC = 0.795, 95% CI [0.753, 0.838]), again closely tracking the 20-item parent scale. Youden-optimal thresholds were ≥ 16 for the STAI-T-6 in both samples (Sample 2: sensitivity 0.679, specificity 0.865, positive predictive value 0.687, negative predictive value 0.861) and ≥ 13 and ≥ 12 for the STAI-S-6 in Samples 2 and 1, respectively. The consistency of the ≥16 threshold for the STAI-T-6 across samples and criteria is encouraging, but all cut-offs reported here should be regarded as provisional pending replication. Repeating these analyses with the Turkish GAD-7 cut-off score of 8 or above (Konkan et al., 2013) did not significantly alter the AUC of either the abbreviated forms or the original 20-item scales; these findings are presented in Supplementary Tables S2,S3 and Supplementary Figure S1.

**Table 8:** Diagnostic accuracy of the abbreviated and the 20-item scales against external criteria.

Sample	Criterion	Measure	AUC [95% CI]	Cut-off	Sens.	Spec.	PPV / NPV
Sample 2 (*n* = 277)	GAD-7 ≥ 10 (84 positive)	STAI-S-6	0.719 [0.655, 0.782]	≥13	0.750	0.585	0.441/0.843
		STAI-S-20	0.756 [0.696, 0.817]	≥46	0.690	0.699	0.500/0.839
		STAI-T-6	0.846 [0.796, 0.897]	≥16	0.679	0.865	0.687/0.861
		STAI-T-20	0.844 [0.794, 0.894]	≥47	0.845	0.746	0.592/0.917
Sample 1 (*n* = 420)	GAD-7 ≥ 10 (192 positive)	STAI-S-6	0.795 [0.753, 0.838]	≥12	0.833	0.627	0.653/0.817
		STAI-S-20	0.811 [0.770, 0.852]	≥41	0.818	0.689	0.689/0.818
		STAI-T-6	0.824 [0.785, 0.863]	≥16	0.708	0.785	0.735/0.762
		STAI-T-20	0.852 [0.817, 0.888]	≥46	0.906	0.662	0.693/0.893

AUC = area under the receiver operating characteristic curve, with 95% confidence intervals computed by the DeLong method; all AUCs differed significantly from 0.50 (*p* < 0.001). Cut-offs maximize the Youden index within the sample in which they were estimated and are therefore provisional. Sens. = sensitivity; Spec. = specificity; PPV = positive predictive value; NPV = negative predictive value. Predictive values depend on the prevalence of the criterion in each sample and will differ in settings with a different base rate.

**Figure 3:**
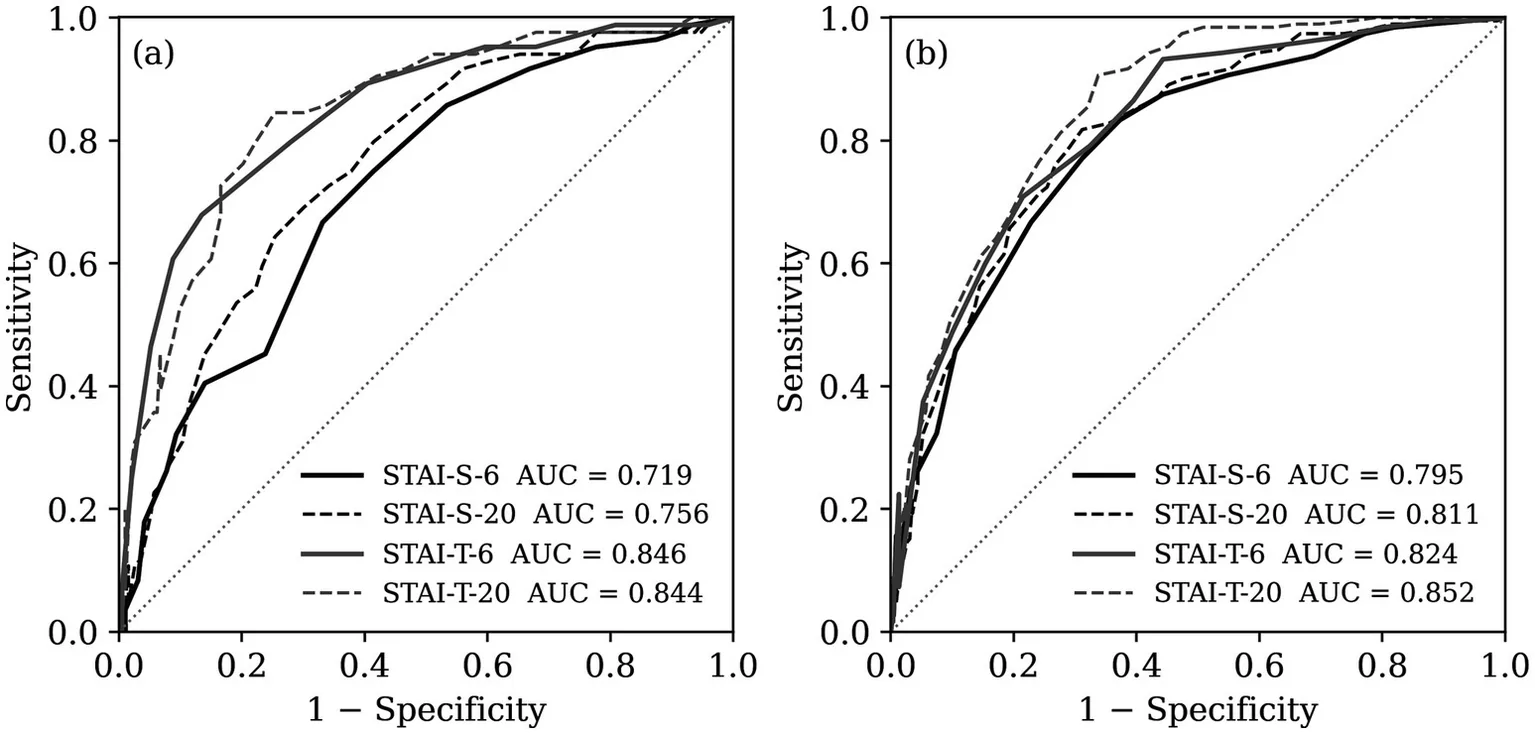
Receiver operating characteristic curves for the abbreviated and full-length STAI scales against a GAD-7 score of 10 or above. **(a)** Sample 2, the school-based cohort (*n* = 277, 84 participants at or above the criterion). **(b)** Sample 1, the clinical cohort (*n* = 420, 192 participants at or above the criterion). Solid lines denote the six-item abbreviated forms and dashed lines the corresponding 20-item parent scales; black lines denote State scales and grey lines Trait scales. The diagonal reference line represents chance-level discrimination. Areas under the curve with confidence intervals, optimal thresholds, and the corresponding sensitivity, specificity, and predictive values are reported in Table 8.

## Discussion

Given the growing demand for efficient assessment instruments in both research and clinical settings, the present study sought to develop abbreviated versions of the STAI-S and STAI-T for use among Turkish adolescents. The findings demonstrated that the six-item versions successfully reduced the length of the original 20-item scales by 70% while preserving their core psychometric properties. This substantial reduction enhances feasibility and administrative efficiency without compromising measurement quality. Consistent with recommendations emphasizing preservation of the original factor structure during scale reduction (Smith et al., 2000), the exploratory analyses confirmed that both abbreviated versions retained the two-factor structure observed in the full-length instruments.

The present forms should be evaluated against a now substantial body of abbreviated STAI instruments. Marteau and Bekker (1992) retained six State items selected on corrected item–total correlations in adult women awaiting surgery, reporting a correlation of 0.95 with the parent scale but no Trait counterpart and no confirmatory analysis. Fioravanti-Bastos et al. (2011) derived a six-item Brazilian short form in adults and, like the present study, recovered a two-factor presence/absence structure, with alpha coefficients in the low 0.80 s. Du et al. (2022) developed a short Chinese version in adult respondents, and Valente et al. (2025) validated abbreviated STAI-Y scales in Italian young adults, additionally demonstrating measurement invariance across sex. Zsido et al. (2020) proposed five-item State and Trait forms in Hungarian adults, and it is these forms that Döner et al. (2024) subsequently adapted into Turkish (STAIS-5, STAIT-5), reporting loadings of 0.706–0.835 and 0.694–0.810 and alpha coefficients of 0.838 and 0.837 in 306 community adults aged 18–59 years. The present instruments differ from previous forms in some respects. First, they are the only abbreviated Turkish STAI forms derived and validated in adolescents; the Turkish STAIS-5/STAIT-5 were validated exclusively in adults, and item performance in 11- to 18-year-olds cannot be assumed from adult data. Second, item selection was carried out in a clinically referred adolescent sample and confirmed in an independent community adolescent sample. Third, this study examines measurement invariance of the shortened Turkish STAI forms across clinical and community groups, as well as by sex and age, which is essential evidence before applying the brief instruments in both contexts. Retaining six rather than five items also permits a balanced three-to-three representation of the presence and absence content poles, which the odd-numbered five-item forms cannot achieve; as the alpha coefficients reported by Döner et al. (2024) indicate, this is achieved without any evident cost to internal consistency. The incremental contribution of the present work is therefore developmental specificity and cross-population evidence rather than mere brevity.

The structural validity of the six-item STAI-S and STAI-T was further examined through CFA in an independent sample of public high school students. Although standardized factor loadings of 0.70 or greater are generally considered desirable, loadings above 0.50 are widely regarded as acceptable indicators of item retention (Hair et al., 2019). In the present study, all standardized factor loadings exceeded this minimum criterion, supporting the meaningful contribution of each item to its respective latent construct. With respect to model fit, all indices demonstrated satisfactory performance except for the RMSEA. Previous research has shown that RMSEA may perform inadequately in structural equation models characterized by a limited number of degrees of freedom, often yielding inflated values even when model specification is appropriate (Shi et al., 2022). Consequently, greater emphasis has been recommended for indices such as the SRMR and CFI when evaluating models of this type (Shi et al., 2022). In the current analyses, SRMR values below 0.08 and CFI values above 0.95 provided support for the two-factor structure of both abbreviated scales. We nonetheless emphasize that the RMSEA exceeded our own prespecified criterion of 0.08 and that its 90% confidence interval was inconsistent with close fit. The structural evidence is therefore best characterized as mixed rather than unequivocal, and the two-factor solution should be regarded as supported but provisional. The comparison with alternative models offers reassurance on this point: the unidimensional alternatives fitted very poorly indeed, so the relative superiority of the two-factor solution is not in doubt even if its absolute fit is imperfect. Replication in larger samples, and models with more degrees of freedom, will be required to resolve this ambiguity.

The question of generalizability across settings deserves particular attention, because item selection was carried out in a sample in which 97.9% of participants were receiving psychiatric follow-up. Items that discriminate well among adolescents in treatment might in principle reflect patterns of anxiety, comorbidity, or symptom endorsement specific to clinical populations rather than the construct as expressed in the general school population. Three features of the present results speak against this concern, though they do not eliminate it. First, the confirmatory analyses were conducted in an entirely independent, predominantly non-clinical sample, in which the structure replicated. Second, and more directly, formal tests of measurement invariance, conducted within the ordinal framework appropriate for four-category items, demonstrated equality of both item thresholds and factor loadings across clinical and community status for both scales. Neither the loadings nor the thresholds therefore appear to be artifacts of the clinical derivation sample, and latent means may be compared directly across the two settings. Third, internal consistency, factor structure and criterion-related associations were of comparable magnitude in both cohorts. The one constraint that was not sustained — equality of residual variances for the STAI-T-6 across settings — indicates that item-specific measurement error is somewhat larger in one setting than in the other. Residual invariance is not required for the comparison of latent means or of associations, and this result does not compromise the intended uses of the instruments, although it does suggest that reliability may not be identical in clinical and community settings.

Concurrent validity was evaluated using two complementary approaches. First, the degree of correspondence between the abbreviated instruments and their original versions was examined. The six-item STAI-S demonstrated a very strong correlation with the original 20-item STAI-S (*r* = 0.949, *p* < 0.001), whereas the six-item STAI-T showed a similarly strong association with the full-length STAI-T (*r* = 0.895, *p* < 0.001). These coefficients must, however, be interpreted with caution, because the six retained items are themselves components of the 20-item totals; part–whole overlap inflates such correlations, and they cannot be regarded as independent evidence of validity. The part–whole corrected coefficients against the non-overlapping remainder of each parent scale (0.75 to 0.88 across samples and scales) provide a more defensible index of construct coverage, and the associations with the GAD-7 and the SWLS, which share no items with the STAI, provide the most informative external validation. Second, associations with external measures were examined to evaluate convergent and divergent validity. As expected, both abbreviated scales demonstrated positive correlations with the GAD-7 and negative correlations with the SWLS. These findings are consistent with established theoretical expectations and previous empirical evidence (Zsido et al., 2020; White and Karr, 2025), providing further support for the validity of the abbreviated STAI-S and STAI-T.

The ROC analyses extend these findings from association to classification. The STAI-T-6 discriminated adolescents scoring at or above the conventional GAD-7 threshold, with an area under the curve of 0.85 in the school sample and 0.82 in the clinical sample, statistically indistinguishable from the 20-item scale in both samples. This is the practically important result for a brief instrument: a 70% reduction in length was achieved with no detectable loss of classification accuracy. These figures should be read as evidence that the abbreviated forms behave like their parent scales in identifying adolescents with higher anxiety symptoms, not as evidence that they can substitute for diagnostic assessment.

Reliability was assessed using Cronbach’s alpha (α) and McDonald’s omega (ω), with values exceeding 0.70 generally considered indicative of acceptable internal consistency (George and Mallery, 2016). All reliability coefficients surpassed this criterion for both the six-item STAI-S (*α* = 0.804 and 0.764; *ω* = 0.805 and 0.766) and the six-item STAI-T (*α* = 0.804 and 0.779; *ω* = 0.809 and 0.784). Although these coefficients are necessarily lower than those of the 20-item parent scales, they exceed the values that would be expected for scales of this length given the mean inter-item correlations of the parent instruments, and they are accompanied by composite reliabilities above 0.82 and average variance extracted above 0.61 for every factor. Collectively, these findings support the reliability of the abbreviated instruments for assessing both state and trait anxiety in Turkish adolescents.

## Limitations

Several limitations should be acknowledged. First, no formal content-validity study was undertaken. Items were retained on statistical grounds supplemented by the authors’ judgment of content coverage, but no expert panel was convened, and no content validity index or ratio was computed. The content representativeness of the abbreviated forms therefore rests on the content validity of the parent instrument and on the balanced retention of presence and absence items, and formal content-validation studies with expert raters and adolescent cognitive interviews remain desirable. Second, test–retest reliability was not assessed. This is a particular limitation for the State scale, whose construct is by definition time-varying and for which the temporal stability characteristics of a six-item form cannot be inferred from those of the parent scale. Third, item selection and exploratory analysis were conducted in a sample in which almost all participants were receiving psychiatric care, and although measurement invariance across clinical and community status was supported, the derivation sample is not representative of Turkish adolescents in general. Fourth, both cohorts were recruited in a single province, limiting geographical generalizability, and the school sample was drawn from two schools only. Fifth, the cut-off scores derived here are provisional: they were identified by maximizing the Youden index within the same samples in which they were estimated, which is known to yield optimistic estimates of accuracy, and they were validated against the GAD-7 rather than against a structured diagnostic interview. Sixth, the confirmatory models have only eight degrees of freedom, which limits the precision of the RMSEA and the power of the test of close fit, as reported above. Finally, all measures were self-reported, so shared method variance may have inflated the observed correlations among them.

## Conclusion

Adolescents often face mental health issues like anxiety caused by factors such as poverty, societal expectations, and academic pressure (Sulejmani and Pop-Jordanova, 2025). Consequently, assessing anxiety within this group is important. In this context, the present findings provide preliminary evidence supporting the reliability and structural validity of the six-item STAI-S and STAI-T in Turkish adolescents. Both forms retained the two-factor structure of the parent instruments, demonstrated acceptable internal consistency, showed measurement invariance across clinical and community settings, sex and age, and preserved the criterion-related performance of the full-length scales while reducing administration burden by 70%. At the same time, the structural evidence was mixed, with the RMSEA exceeding the prespecified threshold in both models, and no evaluation of test–retest reliability, formal content validity, or independently validated cut-off scores is yet available. The abbreviated forms therefore appear well suited to research applications where efficiency is at a premium, but they should not at present be used as a basis for individual diagnostic decisions. Future studies should seek to replicate these findings in larger and more diverse populations, including both clinical and non-clinical adult samples. Application of contemporary psychometric approaches, such as item response theory, may provide additional insight into the measurement characteristics of the abbreviated instruments. Furthermore, the establishment of clinically meaningful cutoff values against structured diagnostic interviews, together with test–retest and content-validity studies, may enhance the utility of these scales for identifying individuals with elevated levels of state and trait anxiety.

## Data Availability

The datasets presented in this article are not readily available because the data that support the findings of this study are available from the corresponding author upon reasonable request. Requests to access the datasets should be directed to AAG, asiyearici@hotmail.com.

## References

[ref1] Browne M. W. Cudeck R. (1992). Alternative ways of assessing model fit. Sociol. Methods Res. 21, 230–258. doi: 10.1177/0049124192021002005

[ref2] Chen F. F. (2007). Sensitivity of goodness of fit indexes to lack of measurement invariance. Struct. Equ. Model. 14, 464–504. doi: 10.1080/10705510701301834

[ref3] Chen J. Wang Q. Liang Y. Chen B. Ren P. (2023). Comorbidity of loneliness and social anxiety in adolescents: bridge symptoms and peer relationships. Soc. Sci. Med. 334:116195. doi: 10.1016/j.socscimed.2023.116195, 37690153

[ref4] Cheung G. W. Rensvold R. B. (2002). Evaluating goodness-of-fit indexes for testing measurement invariance. Struct. Equ. Model. 9, 233–255. doi: 10.1207/s15328007sem0902_5

[ref5] Dağlı A. Baysal N. (2016). Yaşam Doyumu Ölçeğinin Türkçe'ye uyarlanması: Geçerlik ve güvenirlik çalışması [adaptation of the satisfaction with life scale into Turkish: the study of validity and reliability]. Elektron. Sos. Bilim. Derg. 15, 1250–1262. doi: 10.17755/esosder.75955

[ref6] de Lijster J. M. Dieleman G. C. Utens E. M. W. J. Dierckx B. Wierenga M. Verhulst F. C. . (2018). Social and academic functioning in adolescents with anxiety disorders: a systematic review. J. Affect. Disord. 230, 108–117. doi: 10.1016/j.jad.2018.01.008, 29407534

[ref7] DeLong E. R. DeLong D. M. Clarke-Pearson D. L. (1988). Comparing the areas under two or more correlated receiver operating characteristic curves: a nonparametric approach. Biometrics 44, 837–845. doi: 10.2307/2531595, 3203132

[ref8] Dickson S. J. Kuhnert R. L. Lavell C. H. Rapee R. M. (2022). Impact of psychotherapy for children and adolescents with anxiety disorders on global and domain-specific functioning: a systematic review and meta-analysis. Clin. Child. Fam. Psychol. Rev. 25, 720–736. doi: 10.1007/s10567-022-00402-7, 35794304 PMC9622529

[ref9] Dickson S. J. Oar E. L. Kangas M. Johnco C. J. Lavell C. H. Seaton A. H. . (2024). A systematic review and meta-analysis of impairment and quality of life in children and adolescents with anxiety disorders. Clin. Child. Fam. Psychol. Rev. 27, 342–356. doi: 10.1007/s10567-024-00484-5, 38782783 PMC11222200

[ref10] Diener E. D. Emmons R. A. Larsen R. J. Griffin S. (1985). The satisfaction with life scale. J. Pers. Assess. 49, 71–75. doi: 10.1207/s15327752jpa4901_13, 16367493

[ref11] Döner S. Efe Y. S. Elmalı F. (2024). Turkish adaptation of the state–trait anxiety inventory short version (STAIS-5, STAIT-5). Int. J. Nurs. Pract. 30:e13304. doi: 10.1111/ijn.13304, 39323115 PMC11608918

[ref12] Du Q. Liu H. Yang C. Chen X. Zhang X. (2022). The development of a short Chinese version of the state-trait anxiety inventory. Front. Psych. 13:854547. doi: 10.3389/fpsyt.2022.854547, 35619610 PMC9128482

[ref13] Dümmler D. Eck S. Hapfelmeier A. Fomenko A. Aktürk Z. Teusen C. . (2025). State-trait anxiety inventory (STAI) for detecting anxiety disorders in adults. Cochrane Database Syst. Rev. 2025:CD015458. doi: 10.1002/14651858.cd015458, 41416518 PMC12715840

[ref14] Finning K. Ukoumunne O. C. Ford T. Danielson-Waters E. Shaw L. Romero De Jager I. . (2019). The association between anxiety and poor attendance at school–a systematic review. Child Adolesc. Ment. Health. 24, 205–216. doi: 10.1111/camh.12322, 32677217

[ref15] Fioravanti-Bastos A. C. M. Cheniaux E. Landeira-Fernandez J. (2011). Development and validation of a short-form version of the Brazilian state-trait anxiety inventory. Psicol. Reflex. Crit. 24, 485–494. doi: 10.1590/s0102-79722011000300009

[ref16] Fornell C. Larcker D. F. (1981). Evaluating structural equation models with unobservable variables and measurement error. J. Mark. Res. 18, 39–50. doi: 10.1177/002224378101800104

[ref17] George D. Mallery P. (2016). SPSS for Windows Step by Step: A Simple Study Guide and Reference. 14th Edn. New York: Routledge.

[ref18] Hair J. F. Black W. C. Babin B. J. Anderson R. E. (2019). Multivariate data analysis. 8th Edn. Cengage.

[ref19] Hu L. Bentler P. M. (1999). Cutoff criteria for fit indexes in covariance structure analysis: conventional criteria versus new alternatives. Struct. Equ. Model. 6, 1–55. doi: 10.1080/10705519909540118

[ref20] Kaeppler A. K. Erath S. A. (2017). Linking social anxiety with social competence in early adolescence: physiological and coping moderators. J. Abnorm. Child Psychol. 45, 371–384. doi: 10.1007/s10802-016-0173-5, 27282759

[ref21] Kajastus K. Haravuori H. Kiviruusu O. Marttunen M. Ranta K. (2024). Associations of generalized anxiety and social anxiety with perceived difficulties in school in the adolescent general population. J. Adolesc. 96, 291–304. doi: 10.1002/jad.12275, 37985185

[ref22] Kline P. (2000). Handbook of Psychological Testing. 2nd Edn Milton Park, Abingdon: Routledge.

[ref23] Konkan R. Senormanci O. Guclu O. Aydin E. Sungur M. Z. (2013). Validity and reliability study for the Turkish adaptation of the generalized anxiety Disorder-7 (GAD-7) scale. Noro Psikiyatr. Ars. 50, 53–58. doi: 10.4274/npa.y6308

[ref24] MacCallum R. C. Browne M. W. Sugawara H. M. (1996). Power analysis and determination of sample size for covariance structure modeling. Psychol. Methods 1, 130–149. doi: 10.1037/1082-989x.1.2.130

[ref25] Marteau T. M. Bekker H. (1992). The development of a six-item short-form of the state scale of the Spielberger state—trait anxiety inventory (STAI). Br. J. Clin. Psychol. 31, 301–306. doi: 10.1111/j.2044-8260.1992.tb00997.x, 1393159

[ref26] Millsap R. E. Yun-Tein J. (2004). Assessing factorial invariance in ordered-categorical measures. Multivar. Behav. Res. 39, 479–515. doi: 10.1207/s15327906mbr3903_4

[ref27] Öner N. Le Compte A. (1998). Süreksiz Durumluk/Sürekli Kaygı Envanteri El Kitabı. 2. Basım. Boğaziçi Üniversitesi Yayınları: İstanbul [in Turkish].

[ref28] Pityaratstian N. Prasartpornsirichoke J. (2023). Does anxiety symptomatology affect bullying behavior in children and adolescents with ADHD? Child Youth Care Forum 52, 85–103. doi: 10.1007/s10566-022-09681-1

[ref29] Polanczyk G. V. Salum G. A. Sugaya L. S. Caye A. Rohde L. A. (2015). Annual research review: a meta-analysis of the worldwide prevalence of mental disorders in children and adolescents. J. Child Psychol. Psychiatry 56, 345–365. doi: 10.1111/jcpp.12381, 25649325

[ref30] Pollard J. Reardon T. Williams C. Creswell C. Ford T. Gray A. . (2023). The multifaceted consequences and economic costs of child anxiety problems: a systematic review and meta-analysis. JCPP Adv. 3:e12149. doi: 10.1002/jcv2.12149, 37720587 PMC10501703

[ref31] Shi D. DiStefano C. Maydeu-Olivares A. Lee T. (2022). Evaluating SEM model fit with small degrees of freedom. Multivariate Behav Res. 57, 179–207. doi: 10.1080/00273171.2020.1868965, 33576257

[ref32] Smith G. T. McCarthy D. M. Anderson K. G. (2000). On the sins of short-form development. Psychol. Assess. 12, 102–111. doi: 10.1037/1040-3590.12.1.102, 10752369

[ref33] Spielberger C. D. Gorsuch R. L. Lushene R. Vagg P. R. Jacobs G. A. (1983). Manual for the State-Trait Anxiety Inventory. Palo Alto, CA: Consulting Psychologists Press.

[ref34] Spitzer R. L. Kroenke K. Williams J. B. W. Löwe B. (2006). A brief measure for assessing generalized anxiety disorder: the GAD-7. Arch. Intern. Med. 166, 1092–1097. doi: 10.1001/archinte.166.10.1092, 16717171

[ref35] Sulejmani E. Pop-Jordanova N. (2025). Not just numbers: exploring the inner landscape of youth anxiety and stress. Pril (Makedon Akad Nauk Umet Odd Med Nauki). 46, 27–42. doi: 10.2478/prilozi-2025-0020, 41363925

[ref36] Svetina D. Rutkowski L. Rutkowski D. (2020). Multiple-group invariance with categorical outcomes using updated guidelines: an illustration using Mplus and the lavaan/semTools packages. Struct. Equ. Model. 27, 111–130. doi: 10.1080/10705511.2019.1602776

[ref37] Tavakol M. Dennick R. (2011). Making sense of Cronbach's alpha. Int. J. Med. Educ. 2, 53–55. doi: 10.5116/ijme.4dfb.8dfd, 28029643 PMC4205511

[ref38] Valente G. Diotaiuti P. Corrado S. Tosti B. Zanon A. Mancone S. (2025). Validity and measurement invariance of abbreviated scales of the state-trait anxiety inventory (STAI-Y) in a population of Italian young adults. Front. Psychol. 16:1443375. doi: 10.3389/fpsyg.2025.1443375, 39989625 PMC11842307

[ref39] White A. E. Karr J. E. (2025). Psychometric properties of the GAD-7 among college students: reliability, validity, factor structure, and measurement invariance. Transl Issues Psychol Sci. 11, 321–333. doi: 10.1037/tps0000382, 40693694 PMC12221267

[ref40] Wu H. Estabrook R. (2016). Identification of confirmatory factor analysis models of different levels of invariance for ordered categorical outcomes. Psychometrika 81, 1014–1045. doi: 10.1007/s11336-016-9506-0, 27402166 PMC5458787

[ref41] Zsido A. N. Teleki S. A. Csokasi K. Rozsa S. Bandi S. A. (2020). Development of the short version of the Spielberger state—trait anxiety inventory. Psychiatry Res. 291:113223. doi: 10.1016/j.psychres.2020.113223, 32563747

