# Supplementary material for: Measuring state and trait anxiety in Turkish adolescents: development and validation of the six-item short forms of the state–trait anxiety inventory
Source: Front Psychol. 2026 Oct 9;17:1955613. doi: 10.3389/fpsyg.2026.1955613 (PMC13690722; doi:10.3389/fpsyg.2026.1955613)
Supplement: Supplementary file 1 [file Supplementary_file_1.docx]

**Supplementary Material**

**Measuring State and Trait Anxiety in Turkish Adolescents: Development and Validation of the 6-Item Short Forms of the State-Trait Anxiety Inventory**

**Table S1.** Sensitivity analysis: tests of measurement invariance for the 6-item STAI-S and STAI-T estimated by maximum likelihood with the item scores treated as continuous (combined sample, N = 697)

| **Grouping / Model** | **χ²** | **df** | **CFI** | **TLI** | **RMSEA** | **SRMR** | **Δχ² (Δdf)** | **ΔCFI** | **ΔRMSEA** |
| --- | --- | --- | --- | --- | --- | --- | --- | --- | --- |
| **STAI-S-6: clinical (n = 420) vs community (n = 277)** | | | | | | | | | |
| Configural | 29.96 | 16 | .989 | .980 | .050 | .039 | — | — | — |
| Metric | 37.69 | 20 | .986 | .979 | .050 | .044 | 7.73 (4), p = .102 | −.003 | +.000 |
| Scalar | 46.41 | 24 | .983 | .978 | .052 | .044 | 8.72 (4), p = .068 | −.004 | +.001 |
| **STAI-T-6: clinical vs community** | | | | | | | | | |
| Configural | 44.23 | 16 | .980 | .962 | .071 | .046 | — | — | — |
| Metric | 46.08 | 20 | .981 | .972 | .061 | .047 | 1.86 (4), p = .762 | +.002 | −.010 |
| Scalar | 64.42 | 24 | .971 | .964 | .070 | .047 | 18.34 (4), p = .001 | −.010 | +.008 |
| Partial scalar (Item 15 free) | 50.39 | 23 | .980 | .974 | .060 | .047 | 4.31 (3), p = .230 | −.001 | −.001 |
| **STAI-S-6: girls (n = 383) vs boys (n = 314)** | | | | | | | | | |
| Configural | 41.32 | 16 | .980 | .962 | .067 | .049 | — | — | — |
| Metric | 41.66 | 20 | .983 | .974 | .056 | .050 | 0.34 (4), p = .987 | +.003 | −.012 |
| Scalar | 48.50 | 24 | .981 | .976 | .054 | .050 | 6.84 (4), p = .144 | −.002 | −.002 |
| **STAI-T-6: girls vs boys** | | | | | | | | | |
| Configural | 41.96 | 16 | .981 | .964 | .068 | .045 | — | — | — |
| Metric | 43.01 | 20 | .983 | .975 | .057 | .046 | 1.04 (4), p = .903 | +.002 | −.011 |
| Scalar | 45.95 | 24 | .984 | .980 | .051 | .047 | 2.94 (4), p = .567 | +.001 | −.006 |
| **STAI-S-6: age ≤ 14 vs > 14 years** | | | | | | | | | |
| Configural | 34.40 | 16 | .985 | .972 | .057 | .042 | — | — | — |
| Metric | 41.13 | 20 | .983 | .974 | .055 | .046 | 6.73 (4), p = .151 | −.002 | −.002 |
| Scalar | 42.78 | 24 | .985 | .981 | .047 | .045 | 1.64 (4), p = .801 | +.002 | −.008 |
| **STAI-T-6: age ≤ 14 vs > 14 years** | | | | | | | | | |
| Configural | 44.64 | 16 | .979 | .962 | .072 | .047 | — | — | — |
| Metric | 47.45 | 20 | .980 | .971 | .063 | .046 | 2.80 (4), p = .591 | +.001 | −.009 |
| Scalar | 51.56 | 24 | .980 | .975 | .057 | .046 | 4.11 (4), p = .391 | +.000 | −.005 |

*Note.* These analyses are reported for comparability with the earlier literature and are not the basis of any conclusion in the manuscript; the primary invariance analyses treat the four-category items as ordered-categorical and are reported in Table 7. Multiple-group confirmatory factor analyses were estimated by normal-theory maximum likelihood on the observed item scores, with the covariance and mean structures modelled jointly. Configural = loadings and intercepts free in both groups; Metric = loadings constrained equal; Scalar = loadings and intercepts constrained equal, with latent means free in the second group. Each comparison is against the immediately preceding, less restricted model. Invariance was retained when ΔCFI ≤ .010 and ΔRMSEA ≤ .015 in absolute value. Under this continuous approximation, scalar invariance was not supported for the STAI-T-6 across clinical and community status; freeing the intercept of Item 15 established partial scalar invariance. This result was not reproduced in the ordinal analyses reported in Table 7.

**Table S2.** Sensitivity analysis: diagnostic accuracy of the abbreviated and the 20-item scales under two definitions of the GAD-7 criterion

|  | | **Criterion: GAD-7 ≥ 10 (original)** | | | **Criterion: GAD-7 ≥ 8 (Turkish validation)** | | |
| --- | --- | --- | --- | --- | --- | --- | --- |
| **Sample** | **Measure** | **AUC [95% CI]** | **Cut-off** | **Sens. / Spec.** | **AUC [95% CI]** | **Cut-off** | **Sens. / Spec.** |
| **Sample 2 (school-based, n = 277): 84 positive (30.3%) at ≥ 10, 119 positive (43.0%) at ≥ 8** | | | | | | | |
|  | STAI-S-6 | .719 [.655, .782] | ≥ 13 | .750 / .585 | .739 [.681, .798] | ≥ 13 | .731 / .646 |
|  | STAI-S-20 | .756 [.696, .817] | ≥ 46 | .690 / .699 | .781 [.726, .835] | ≥ 44 | .739 / .696 |
|  | STAI-T-6 | .846 [.796, .897] | ≥ 16 | .679 / .865 | .827 [.779, .875] | ≥ 14 | .832 / .658 |
|  | STAI-T-20 | .844 [.794, .894] | ≥ 47 | .845 / .746 | .833 [.784, .881] | ≥ 46 | .765 / .759 |
| **Sample 1 (clinical, n = 420): 192 positive (45.7%) at ≥ 10, 228 positive (54.3%) at ≥ 8** | | | | | | | |
|  | STAI-S-6 | .795 [.753, .838] | ≥ 12 | .833 / .627 | .793 [.749, .836] | ≥ 13 | .737 / .734 |
|  | STAI-S-20 | .811 [.770, .852] | ≥ 41 | .818 / .689 | .814 [.772, .855] | ≥ 42 | .750 / .771 |
|  | STAI-T-6 | .824 [.785, .863] | ≥ 16 | .708 / .785 | .822 [.782, .862] | ≥ 14 | .838 / .661 |
|  | STAI-T-20 | .852 [.817, .888] | ≥ 46 | .906 / .662 | .854 [.818, .891] | ≥ 46 | .864 / .719 |

*Note.* The main analyses (Table 8) used a GAD-7 score of 10 or above, the threshold recommended in the original development study. Because the Turkish validation of the GAD-7 identified 8 as the most acceptable cut-off point in a Turkish clinical sample (19), all receiver operating characteristic analyses were repeated using that threshold. AUC = area under the curve, with 95% confidence intervals computed by the DeLong method; all AUCs differed significantly from .50 (p < .001). Cut-offs maximise the Youden index within the sample in which they were estimated. Sens. = sensitivity; Spec. = specificity. Differences in AUC between the two criterion definitions were tested by nonparametric bootstrap (4,000 resamples) and are reported, together with DeLong comparisons of the abbreviated and 20-item forms, in Table 9 of the main text; no AUC differed significantly between the two criteria (all p ≥ .28).

**Table S3.** Performance of the cut-off scores retained in the manuscript under both definitions of the GAD-7 criterion

| **Sample** | **Measure (cut-off)** | **Criterion** | **Sens.** | **Spec.** | **PPV** | **NPV** |
| --- | --- | --- | --- | --- | --- | --- |
| Sample 2 | STAI-S-6 (≥ 13) | GAD-7 ≥ 10 | .750 | .585 | .441 | .843 |
|  |  | GAD-7 ≥ 8 | .731 | .646 | .608 | .761 |
|  | STAI-T-6 (≥ 16) | GAD-7 ≥ 10 | .679 | .865 | .687 | .861 |
|  |  | GAD-7 ≥ 8 | .563 | .899 | .807 | .732 |
| Sample 1 | STAI-S-6 (≥ 12) | GAD-7 ≥ 10 | .833 | .627 | .653 | .817 |
|  |  | GAD-7 ≥ 8 | .794 | .667 | .739 | .731 |
|  | STAI-T-6 (≥ 16) | GAD-7 ≥ 10 | .708 | .785 | .735 | .762 |
|  |  | GAD-7 ≥ 8 | .667 | .828 | .822 | .677 |

*Note.* PPV = positive predictive value; NPV = negative predictive value. Predictive values depend on the prevalence of the criterion, which is higher under the ≥ 8 definition (43.0% in Sample 2 and 54.3% in Sample 1) than under the ≥ 10 definition (30.3% and 45.7%); positive predictive values therefore rise and negative predictive values fall when the more inclusive threshold is applied, independently of any change in the discriminative ability of the STAI forms.

**Figure S1.** Receiver operating characteristic curves for the abbreviated and the 20-item STAI scales against a Generalized Anxiety Disorder-7 score of 8 or above, the cut-off established in the Turkish validation of the GAD-7

*Note.* (a) Sample 2, the school-based cohort (n = 277, 119 participants at or above the criterion). (b) Sample 1, the clinical cohort (n = 420, 228 participants at or above the criterion). Solid lines denote the 6-item abbreviated forms and dashed lines the corresponding 20-item parent scales; black lines denote State scales and grey lines Trait scales. The diagonal reference line represents chance-level discrimination.

*Interpretation.* Adopting the Turkish GAD-7 threshold of 8 rather than the internationally recommended threshold of 10 leaves the discriminative ability of the abbreviated forms essentially unchanged. Across the eight comparisons in Table S2 the area under the curve shifts by no more than .025 (STAI-S-20 in Sample 2) and by only .020 and .002 for the STAI-T-6, every confidence interval under one criterion contains the point estimate obtained under the other, and formal bootstrap tests showed that none of these differences was statistically significant (all p ≥ .28; Table 9 of the main text). The relation between the abbreviated and full-length forms is also unchanged: the STAI-T-6 does not differ from its 20-item parent scale in Sample 2 (.827 vs .833, p = .690) and remains modestly lower in Sample 1 (.822 vs .854, p = .006), exactly as under the original criterion, and the ordering of the four measures is preserved in both cohorts. Because the area under the curve is a threshold-free index, this is the expected result if the abbreviated forms measure the same dimension of anxiety irrespective of where the criterion is drawn, and it indicates that the conclusions of the main analysis do not depend on the choice of GAD-7 threshold.

What does change is the Youden-optimal cut-off for the STAI-T-6, which falls from 16 to 14 in both samples. This is a direct consequence of the criterion becoming more inclusive: the ≥ 8 definition identifies a larger and, on average, less severely anxious group (43.0% rather than 30.3% of Sample 2), so the threshold that best balances sensitivity and specificity moves downward. The trade-off is visible in Table S3. At ≥ 14 the STAI-T-6 detects 83% of adolescents scoring at or above GAD-7 ≥ 8 at the cost of a specificity of .66, whereas at ≥ 16 it retains a specificity of .90 with a sensitivity of .56. The optimal cut-off for the STAI-S-6 is by contrast almost unaffected (≥ 13 under both criteria in Sample 2, and ≥ 12 versus ≥ 13 in Sample 1). Practically, these results suggest that ≥ 14 is the more appropriate threshold for the STAI-T-6 when the aim is inclusive first-stage screening against the Turkish GAD-7 criterion, and ≥ 16 when a higher specificity is required, for instance to limit false positives in settings with restricted follow-up capacity. They also reinforce the caution stated in the main text: cut-off scores are criterion-dependent and remain provisional until they are validated against a structured diagnostic interview in an independent sample.
